# Incidence of Central Line-Associated Bloodstream Infection in a Tertiary Care Hospital in Northern India: A Prospective Study

**DOI:** 10.7759/cureus.44501

**Published:** 2023-08-31

**Authors:** Safia Maqbool, Rajni Sharma

**Affiliations:** 1 Microbiology, Sawai Man Singh (SMS) Medical College and Hospital, Jaipur, IND

**Keywords:** blood stream infection (bsi), central venous catheter, intensive care unit, central line-associated bloodstream infection, s: hospital acquired infection

## Abstract

Background

Central line-associated bloodstream infection is the most common hospital-acquired infection and is associated with high morbidity and mortality along with increased healthcare cost. However, studies on the incidence of nosocomial infections are very limited in India.

Aims

To determine the incidence of central line-associate bloodstream infection (CLABSI), microorganisms associated and their antimicrobial sensitivity profile in the medical ICU of a tertiary care hospital.

Material and methods

A total of 186 patients who were admitted to the medical ICU and had a non-tunneled central venous catheter (CVC) implanted at admission in the emergency department or in the medical ICU for longer than 48 hours were monitored. By examining the blood culture reports, the patients were monitored every day for the emergence of new-onset sepsis after 48 hours following CVC insertion. The data were evaluated statistically using Microsoft Excel and SPSS version 22.0 (IBM Corp., Armonk, NY, USA).

Result

Out of 186 catheterized patients, 37 developed CLABSI. The incidence of CLABSI was 9.3 per 1000 catheter days and 6.7 per 1000 inpatient days with a 0.7 device utilization ratio. The most common organism isolated was *Acinetobacter species *(22%) followed by *K. pneumoniae* (16%) and *E. aerogenes* (16%). The highest sensitivity was displayed by polymyxin B (100%) followed by tigecycline (85.48%) and minocycline (50.82%) in Gram-negative organisms. In Gram-positive organisms, the highest sensitivity was observed in *S. aureus* (100%) for vancomycin, linezolid and teicoplanin whereas *Enterococcus species* showed linezolid (100%) followed by vancomycin (93.75%) and teicoplanin (93.75%).

Conclusion

The prevention of CLABSI requires knowledge of the infection rates and of the sources, the pathogens involved as well as their antimicrobial profile. Due to rising antimicrobial resistance, surveillance programs are crucial in establishing the species distribution and resistance patterns of bacteria causing BSIs and thus providing the basis for appropriate empirical therapy.

## Introduction

Hospital-acquired infection is a most complicated public health issue which affects millions of people worldwide and is the most common complication in ICU patients as they are related to high morbidity and mortality [1]. Central line-associated bloodstream infection (CLABSI) is the most common healthcare-associated infection (HAI) in which the infection occurs as a result of pathogens entering the bloodstream through a central venous catheter [2-3]. The adult population mostly with comorbidities is at higher risk [4] and is the leading cause of morbidity and mortality in hospitals worldwide. Central venous catheter (CVC) is used to manage critically ill patients who need long-term intravenous medicine, nutritional support, hemodynamic monitoring, plasmapheresis, and haemodialysis, as well as the provision of fluids, pharmaceuticals, and blood products for infusion therapy. The most avoidable type of nosocomial infection is CLABSI [5]. In low-income countries, the rate of CLABSI for adults ranged from 1.6 to 44.6/1000 catheter days as compared to the United States (1.5/1000 catheter days) [6].

Microorganisms-associated CLABSI range from virulent microorganisms to the normal resident microbiome of the skin at the insertion site. Acinetobacter spp., Klebsiella spp., Candida spp., Staphylococcus aureus, Enterococcus spp., and coagulase-negative Staphylococcus have been the most prevalent species associated with CLABSI [7-11]. There is also a high incidence of multidrug resistance (MDR) related to CLABSI [12].

## Materials and methods

Study design

This hospital-based prospective study was conducted in the Department of Microbiology and Medical ICU (MICU) of SMS Medical College and attached Hospitals, Jaipur, Rajasthan. The study was approved by the Institutional Ethics Committee and Research Review Board of SMS Medical College and Hospital with IRB number 281MC/EC/2021.

Methodology

Patients fulfilling the inclusion criteria were enrolled in this study. Written and informed consent was obtained from each patient. The inclusion criteria included adult patients >18 years of age with a central venous catheter inserted at the Emergency Department or in the Medical ICU of our hospital for >48 hours. The exclusion criteria included patients showing positive blood culture or clinical signs or symptoms of infection like fever etc. at the time of admission or < 48 hrs of admission to the surveillance unit, patients admitted with indwelling central venous catheter in place from other hospital and a single commensal identified in a single blood specimen (contaminant). With a 95% confidence interval and 5% margin of error, the sample size was 186 patients.

A site-specific prospective active surveillance was carried out on a daily basis in MICU and the detailed history of each patient fulfilling the inclusion criteria was collected and denominator data was also collected daily as per the guidelines of CDC NHSN. The patient details like patient name, medical record number, hospital and ICU admission date; demographic data like gender, age, medical history and co-morbidities and admission diagnosis; and insertion of invasive devices (such as central venous catheter) along with the purpose of intervention and date of insertion and removal were collected. For calculating the incidence of CLABSI monthly denominator data was recorded daily for MICU - it included patient-day (total number of patients per day in MICU) and central-line days (number of patients with one or more temporary central lines in MICU, each day).

Criteria for defining CLABSI according to CDC NHSN

CLABSI was defined as a primary laboratory-confirmed bloodstream infection if a recognized pathogen was grown from one or more percutaneous blood cultures after 48 hours of vascular catheterization and the pathogen was unrelated to an infection at another location. And if common skin commensals, such as diphtheroids, Bacillus species, Propionibacterium species, coagulase-negative staphylococci, or micrococci, were cultivated from two or more blood cultures taken on different occasions along with at least one of the following signs or symptoms: Fever (>38° C) or hypotension [13].

Daily monitoring for the onset of infection in terms of clinical signs and symptoms was performed. With the clinical suspicion of sepsis, laboratory work-ups were carried out to identify the other source of infection. The onset of infection was suspected when at least two of the following conditions were present along with suspicion of the sepsis: fever (>38° C), tachycardia (>90 beats per minute) or tachypnoea (>24 breaths per minute) and leukocytosis (>12000/mm^3^) or leukopenia (<4000/mm^3^). Blood specimens from such patients were drawn from peripheral venipuncture/lumen of central line as per standard laboratory protocol and sent to the Microbiology department for culture and sensitivity testing. To exclude the other sources of infection physical examination and investigations like urine cultures, sputum cultures, tracheal aspirates, and imaging reports were performed depending on the clinical profile of the patient. If no other source of infection was found, then the sepsis was suspected.

Sample processing

Blood culture vials were loaded in the automated blood culture system BACT/ALERT 3D (Biomerieux, USA) and incubated at 37°C for up to five days. Positive blood culture vials were subcultured by qualitative method on Blood agar and MacConkey agar [14-15] and microbial growth was then identified conventionally by Gram stain, Colony morphology and various biochemical tests as per Standard laboratory protocol [16]. Antimicrobial susceptibility testing was done by Kirby-Bauer Disc diffusion method on Muller Hinton agar (MHA) according to Clinical and Laboratory Standard Institute (CLSI) guidelines, 2019 [17].

Calculation of incidence

CLABSI rate was calculated by the formula, total number of reported CLABSI/number of central line days multiplied by 1000 and Device Utilization Ratio (DUR) was calculated by formula, number of device days/number of patient days [13].

Statistical analysis

Statistical analysis was performed using percentage, mean, median and standard deviation. Statistical data was compiled, tabulated and examined statistically using SPSS version 22.0 (IBM Corp., Armonk, NY, USA) to obtain valid results.

## Results

During the study period, a total of 186 patients fulfilled the inclusion criteria and were enrolled in the study. Out of these 37 patients developed central line-associated bloodstream infection, accounting for 3994 catheter days and 5389 inpatient days. Thus, the incidence of CLABSI was 9.3 per 1000 central line days and 6.7 per 1000 inpatient days with a 0.7 device utilization ratio as shown in Table 1.

**Table 1:** Central line-associated bloodstream infection (CLABSI) incidence rate The table shows the incidence rate of central line-associated bloodstream infection. The incidence rate of CLABSI was 9.3/1000 catheter days for a 0.7 device utilization ratio. CVC: Central venous catheter

Characteristics of central line	CLABSI
No. of CVC days	3994
No. of inpatient days	5389
CLABSI incidence (per 1000 catheter days)	9.3
Device utilization ratio (DUR)	0.7

**Figure 1:**
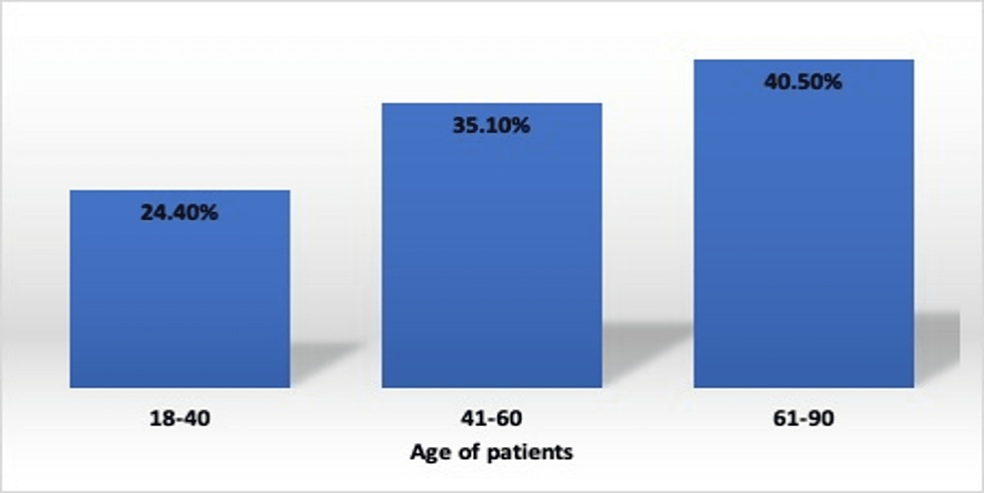
Frequency of patients with central line-associated bloodstream infection (CLABSI) in different age groups The figure shows the association of CLABSI with age. The highest rate of CLABSI was observed in the elderly age group 61-90 years.

The incidence of central line-associated bloodstream infection (CLABSI) was higher in males (64.9%) as compared to females (35.1%) as shown in Table 2. We observed an increased incidence of CLABSI in the geriatric age group. The most common age group affected was 61-90 years followed by 41-60 years with a mean of 48.8 ± 20.1 years as shown in Tables 3, 4 and Figure 1.

**Table 2:** Description of central line-associated bloodstream infection rates by patient gender The table shows the gender of patients commonly affected by CLABSI. The rate of CLABSI was higher in males (64.9%) as compared to females (35.1%).

Gender of patients with CLABSI	n (%) Total = 37
Male	24 (64.9%)
Female	13 (35.1%)

**Table 3:** Frequency of patients with central line-associated bloodstream infection (CLABSI) in different age groups The table shows the age group commonly associated with CLABSI. The most common age group associated with CLABSI was 61-90 years with a mean age of 48.8 ± 20.1 years.

Age group of patients (years)	CLABSI n (%) Total = 37
18-40	9 (24.4%)
41-60	13 (35.1%)
61-90	15 (40.5%)

**Table 4:** Descriptive statistics of age of overall patients versus patients with central line-associated bloodstream infection (CLABSI)

Characteristics (Age)	Total patients (n = 186)	CLABSI (n = 37)
Minimum	18	18
Maximum	92	90
Median (IQR)	53 (33.3-67)	51 (28.3-66.8)
Mean ± SD	51.5 ± 20.9	48.8 ± 20.1

**Figure 2:**
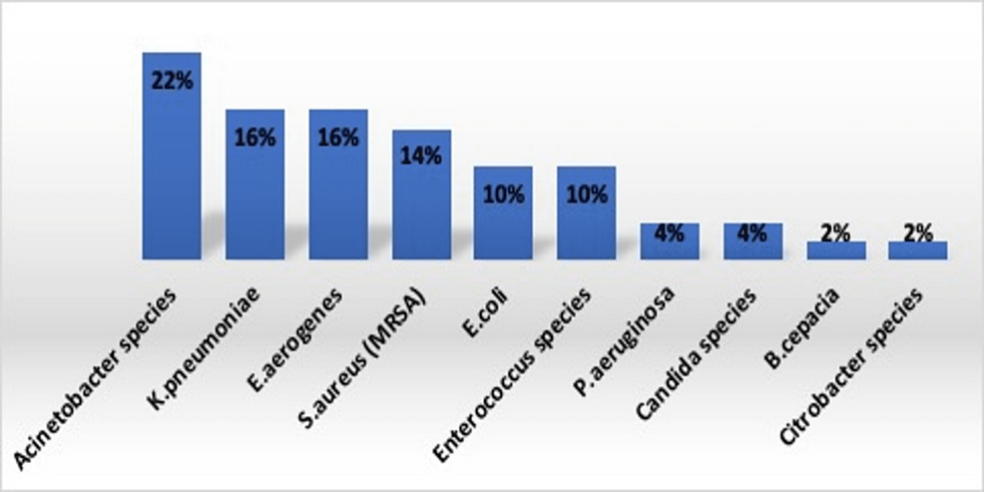
Microbial etiology of patients with central line-associated bloodstream infection (CLABSI) The figure shows the microbial etiology associated with CLABSI. The most common organism associated with CLABSI was *Acinetobacter*​ species followed by *K. pneumoniae* and* E. aerogenes.*

Table 5 shows the microbial etiology of patients with CLABSI. A total of 50 microorganisms were isolated from these 37 CLABSI patients as there was polymicrobial growth in six (12%) blood samples in culture. We observed that Gram-negative organisms were predominant (72%) followed by Gram-positive organisms (24%) and Candida species (4%). The most common organism associated with CLABSI was *Acinetobacter *species (22%) followed by *K. pneumoniae* and *E. aerogenes *(16%) each (Figure 2). In Gram-negative organism, the highest antimicrobial sensitivity was observed in polymyxin B (100%) followed by tigecycline (85.48%) and minocycline (50.82%). All the seven isolates of *Staphylococcus aureus* were resistant to methicillin. In Gram-positive organisms, the highest sensitivity of 100% was displayed by *Staphylococcus aureus* to vancomycin, teicoplanin and linezolid whereas *Enterococcus *species displayed 100% sensitivity to linezolid followed by teicoplanin (80%). Out of five isolates of *Enterococcus* species, one isolate (20%) was vancomycin-resistant.

**Table 5:** Microbial etiology of patients with central line-associated bloodstream infection (CLABSI) The table shows the microbial etiology of patients with CLABSI. The most common organism associated with CLABSI was *Acinetobacter* species followed by *K. pneumoniae* and* E. aerogenes.*

Etiology	CLABSI n (%) Total = 50
Acinetobacter species	11 (22%)
K. pneumoniae	8 (16%)
E. aerogenes	8 (16%)
S. aureus (MRSA)	7 (14%)
E. coli	5 (10%)
Enterococcus species	5 (10%)
P. aeruginosa	2 (4%)
Candida species	2 (4%)
B. cepacia	1 (2%)
Citrobacter species	1 (2%)

The outcome of patients who developed central line-associated bloodstream infection was poor as compared to patients with no blood-stream infection. As shown in Table 6, we observed that the mortality rate (62.2%) was higher in patients with CLABSI as compared to patients with no BSI (58.4%).

**Table 6:** Outcome of patients with and without central line-associated bloodstream infection (CLABSI) The table shows the mortality rate associated with CLABSI. The mortality rate of patients with CLABSI was higher (62.2%) as compared to patients with no BSI (58.4%).

Outcome	Patients with No BSI n (%) Total = 149	Patients with CLABSI n (%) Total = 37
Died	87 (58.4%)	23 (62.2%)
Discharge	56 (37.6%)	12 (32.4%)
LAMA	6 (4%)	2 (5.4%)

## Discussion

Out of all types of nosocomial infections, CLABSIs are the most fatal and associated with costly healthcare. The rates of CLABSI in ICUs of developing countries are higher than the developed countries. The incidence of nosocomial bacteraemia is underestimated, and only sparse information is available from nations with inadequate resources, like India. Therefore, the aim of this study was to determine the incidence of CLABSI, the pathogens involved and their antimicrobial sensitivity pattern in an adult ICU in northern India.

Our study included 186 patients in the age group of >18 years with central venous catheterization for >48 hrs. Out of these, 37 patients developed nosocomial bacteraemia. Thus, the incidence of CLABSI in our study was 9.3 per 1000 central line days and 6.7 per 1000 inpatient days with 0.7 device utilization ratio (Table 1), which was comparable with the incidence reported by the following authors (Table 7).

**Table 7:** Incidence of central line-associated bloodstream infection (CLABSI) per 1000 catheter days

S.No	Author	Year	Place	CLABSI Incidence/1000 CL^* ^days
1	Present study	2023	India	9.3
2	Masih et al. [18]	2016	India	13.35
3	Al-Tawfiq et al. [19]	2013	S. Arabia	10.0
4	Parameswaran et al. [20]	2011	India	8.6
5	Dogru et al. [21]	2010	Turkey	11.8

These rates are higher than developed countries such as an incidence of 1.05 was reported from USA [22]. However, in the WHO region of Europe, the CLABSI rates were lower - Tutuncu et al. reported 2.8 and Yalaz et al. reported 3.8 incidence of CLABSI per 1000 central line days [23, 24]. The reason for the higher incidence of CLABSI in our study might be due to differences in infection control prevention practices, multidrug-resistant pathogens acquired via invasive procedures, inappropriate use of invasive devices, excessive or improper antibiotic use, and low healthcare professional-to-patient ratio. However, a higher incidence was reported by Mishra et al. (17.04), Chopdekar et al. (27.065) and Johnson et al. (29.3) [25-27]. We observed male predominance in CLABSI cases (Table 2). Similar findings of male predominance were reported by Endimiani et al. (72.8%) and Dasgupta et al. (72.4%) [28-29]. The main predisposing factors associated with CLABSI are underlying health status (chronic illness, surgery, trauma), advanced age, invasive procedures and invasive devices [30]. The increased incidence of CLABSI was in the geriatric age group. The most common age group affected was 61-90 years followed by 41-60 years with a mean of 48.8 ± 20.1 years (Table 3). Comparable studies were reported by Endimiani et al. (65 ± 17 years) and Singh et al. (41-60 years with a mean age of 50.93 ± 14.08 years) [28, 31]. The reason for the high incidence of CLABSI in the elderly age group might be that these patients have defective host defence mechanisms, immunosuppression, and higher severity of illness and all these factors might have rendered elderly patients more susceptible to CLABSI.

*Acinetobacter *species have emerged as important nosocomial pathogens, with high resistance to antimicrobials and propensity to survive on environmental surfaces [32]. In the present study, we observed that the most common organism associated with CLABSI was *Acinetobacter* species (22%) followed by *K. pneumoniae* (16%) and *E. aerogenes *(16%) (Table 5), and this finding was comparable to the studies reported by Mathur et al. (21.7%) and Khurana et al. (24.09%) [33, 34]. The reason for the higher rate of Acinetobacter species associated with CLABSI in the present study might be that it is an opportunistic organism and therefore affects immunocompromised patients. The highest antimicrobial sensitivity was displayed by polymyxin B (100%) followed by tigecycline (85.48%) and minocycline (50.82%) by Gram-negative organisms. We observed 100% prevalence of MRSA which was similar to the findings of Tomar et al., who reported 100% MRSA [35]. In contrast to our study, Tolera et al. reported 88.9% MRSA [36]. In Gram-positive organisms, the highest sensitivity of 100% was displayed by *S. aureus* to vancomycin, teicoplanin and linezolid whereas Enterococcus species displayed sensitivity to linezolid (100%) followed by teicoplanin (80%). In India, the prevalence of VRE is in increasing trend. In our study, we found only one (20%) isolate of vancomycin-resistant Enterococcus species (VRE). The common risk factors for VRE bacteraemia are prolonged ICU stay, immunosuppression, surgeries and overuse of antibiotics. It is necessary to identify the VRE strains and take preventive infection control measures to limit the spread of VRE, which can lead to serious consequences. Nosocomial infections are associated with increased morbidity and mortality. We observed the highest mortality rate in patients with CLABSI (62.2%) as compared to patients with no BSI (58.4%). Our findings were comparable with the study of Mishra et al., who reported 56% mortality in CLABSI patients as compared to an overall mortality of 46% [25].

Our study provided the prospective incidence of CLABSI along with microbial etiology and antimicrobial sensitivity profile associated with CLABSI in an adult medical ICU in northern India. However, there were some limitations in our study such as data regarding the preventive bundle care measures during central venous catheter insertion, access, and maintenance.

## Conclusions

In our study, the incidence of CLABSI was higher than the developed nations and the pathogens associated with CLABSI were multi-drug resistant. The prevention of CLABSI requires knowledge of the infection rates and of the sources, the pathogens involved as well as their antimicrobial profile. Due to rising antimicrobial resistance, surveillance programs are crucial in establishing the species distribution and resistance patterns of bacteria causing BSIs and thus provide the basis for appropriate empirical therapy. Therefore, surveillance programmes should be encouraged which will help in reducing these nosocomial infections and thus ultimately help in better outcomes for patients.
